# Dissection, in vivo imaging and analysis of the mouse epitrochleoanconeus muscle

**DOI:** 10.1111/joa.13478

**Published:** 2021-06-13

**Authors:** David Villarroel‐Campos, Giampietro Schiavo, James N. Sleigh

**Affiliations:** ^1^ Department of Neuromuscular Diseases UCL Queen Square Institute of Neurology University College London London UK; ^2^ UK Dementia Research Institute University College London London UK

**Keywords:** amyotrophic lateral sclerosis, axonal transport, Charcot‐Marie‐Tooth disease, live imaging, motor neuron, muscle fibre type, neuromuscular junction, spinal muscular atrophy

## Abstract

Analysis of rodent muscles affords an opportunity to glean key insights into neuromuscular development and the detrimental impact of disease‐causing genetic mutations. Muscles of the distal leg, for instance the gastrocnemius and tibialis anterior, are commonly used in such studies with mice and rats. However, thin and flat muscles, which can be dissected, processed and imaged without major disruption to muscle fibres and nerve‐muscle contacts, are more suitable for accurate and detailed analyses of the peripheral motor nervous system. One such wholemount muscle is the predominantly fast twitch epitrochleoanconeus (ETA), which is located in the upper forelimb, innervated by the radial nerve, and contains relatively large and uniformly flat neuromuscular junctions (NMJs). To facilitate incorporation of the ETA into the experimental toolkit of the neuromuscular disease field, here, we describe a simple method for its rapid isolation (<5 min), supported by high‐resolution videos and step‐by‐step images. Furthermore, we outline how the ETA can be imaged in live, anaesthetised mice, to enable examination of dynamic cellular processes occurring at the NMJ and within intramuscular axons, including transport of organelles, such as mitochondria and signalling endosomes. Finally, we present reference data on wild‐type ETA fibre‐type composition in young adult, male C57BL6/J mice. Comparative neuroanatomical studies of different muscles in rodent models of disease can generate critical insights into pathogenesis and pathology; dissection of the wholemount ETA provides the possibility to diversify the repertoire of muscles analysed for this endeavour.

## Graphical abstract

The mouse epitrochleoanconeus (ETA) is a thin and flat muscle located in the upper forelimb. Aided by step‐by‐step images and videos, we describe a simple method for its rapid isolation to analyse muscle fibre types and neuromuscular junctions (NMJs). We also outline how the ETA can be imaged in live, anaesthetised mice to enable examination of dynamic cellular processes occurring at the NMJ and within intramuscular axons.

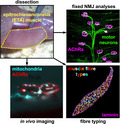

## 1 INTRODUCTION

Rodent models have been instrumental to identifying fundamental features of disease pathogenesis and pathology in a broad range of conditions affecting the neuromuscular system, including amyotrophic lateral sclerosis (ALS) and Charcot‐Marie‐Tooth disease (De Giorgio et al., 2019; Juneja et al., 2019; Sleigh et al., 2011; Webster, 2018). Although there are clear anatomical differences between human and rodent peripheral nerves, for example, axon length, neuromuscular junction (NMJ) and terminal Schwann cell morphology (Al‐hindi et al., 2021; Boehm et al., 2020; Jones et al., 2017) and muscle fibre‐type proportions (Schiaffino & Reggiani, 2011), having a detailed temporal understanding of neuropathology in rodent models of disease is crucial for pathomechanism discovery and evaluation of preclinical therapeutics. Because lower motor neurons are not uniformly vulnerable to disease, comparative analyses of phenotypes, such as impaired NMJ innervation and neurotransmission, across different muscles provide further opportunities for determining key disease processes (Ling et al., 2012; Murray et al., 2008, 2015; Ruiz et al., 2010; Sleigh et al., 2014b; Torres‐Benito et al., 2011). When combined with knowledge of distinctions between wild‐type muscles (e.g., gene expression, receptor protein levels or anatomical features, such as fibre‐type composition and NMJ morphology), correlations with neuropathology can be assessed, and significant contributors to disease may be identified (Boyd et al., 2017; Frey et al., 2000; Kaplan et al., 2014; Kline et al., 2017; Sleigh et al., 2020a; Thomson et al., 2012).

Muscles of the distal leg, for example, gastrocnemius, soleus and tibialis anterior, are frequently chosen for neuromuscular analyses (Burgess et al., 2016). However, while these thick and relatively large muscles are easy to dissect (Oishi et al., 2011), they require sectioning or teasing for neuromuscular analyses, which can be time‐consuming and does not readily allow for assessment of all synapses throughout the muscle. Optical tissue clearing of these muscles can allow muscle‐wide NMJ analysis (Yin et al., 2019), but antibody penetration issues persist (Williams et al., 2019). Alternatively, thin and flat muscles, which can be dissected and immunohistochemically analysed as wholemount preparations without the need for sectioning, provide the major advantage that the entire innervation pattern and NMJ population can be quickly and easily visualised without needing specialised equipment or protocols. Additionally, sectioning artefacts are avoided, while simple and highly reliable immunohistochemical techniques are available for imaging neuromuscular synapses and fibres from wholemount muscles. Although more challenging, helpful dissection protocols and descriptions are available for several wholemount muscles, including cranial, diaphragm, hind paw lumbricals and transversus abdominis (Angaut‐Petit et al., 1987; Murray et al., 2010, 2014; Ojeda et al., 2020; Sleigh et al., 2014a; Wu & Mei, 2013).

Live imaging of biological processes can provide a wealth of information that is either lost or obscured by only working with fixed tissues (Lichtman & Fraser, 2001). For instance, rapid time‐lapse imaging over minutes to hours, as well as repeated, longitudinal in vivo imaging over extended periods, have provided key insights into the neuromuscular synapse during development, ageing and in disease, which would have otherwise been impossible to identify via fixed sample imaging (Li et al., 2011; Martineau et al., 2018; Turney & Lichtman, 2012). Moreover, in vitro measurements of dynamic neuronal processes, such as axonal transport, can differ from those observed in vivo (Gibbs et al., 2016; Sleigh et al., 2017b). Consequently, it is important to be able to study the intact neuromuscular system in its natural environment. Due to accessibility restrictions, intravital NMJ studies have been performed in only relatively few muscles, including the thin and flat sternomastoid muscle in the neck (Li et al., 2011; Lichtman et al., 1987; Turney & Lichtman, 2012) and at superficial synapses of hind (pelvic) limb muscles (Bruusgaard et al., 2003; Hill & Robbins, 1991; Martineau et al., 2018; Mercier et al., 2016; Yampolsky et al., 2010). Unfortunately, protocols outlining these in vivo imaging procedures are rare (Blanco & Ribchester, 2012; Turney et al., 2012).

The epitrochleoanconeus (ETA), also termed the anconeus epitrochlearis, is a thin, roughly rectangular muscle, located in proximity to the triceps brachii on the medial surface of the upper forelimbs that contributes to forearm supination in rodents (i.e., outward paw rotation). Innervated by a branch of the radial nerve containing ~12 axons (Nguyen et al., 2012), the mouse ETA is reported to consist of ~90% fast twitch muscle fibres (Bradley et al., 1989). Blood is supplied to the ETA from the brachial artery via a descending branch of the arteria profunda brachii (Greene, 1935). Not be confused with the anconeus muscle, the ETA is not present in all primates (Diogo et al., 2012; Vanhoof et al., 2020) but is found in ~10–15% of humans (Campbell et al., 1991; Dellon, 1986), where it may protect the unlar nerve from pressure. In rodents, the ETA can be quickly and easily dissected and wholemount processed without the need for sectioning. Consequently, it has been used for electrophysiological recordings (Nanou et al., 2016; Rogozhin et al., 2008; Wang et al., 2018), as well as assessment of pathological changes in NMJ morphology and function in several mouse models of neuromuscular disease (Lyons & Slater, 1991; Nguyen et al., 2012; Sleigh et al., 2020a; Tarr et al., 2013). Given its superficial, accessible position and relatively large, en face NMJs (Mech et al., 2020; van der Pijl et al., 2016), the ETA is also an ideal candidate for intravital imaging.

To increase the diversity of muscles available for comparative analyses both in fixed tissue and in vivo, here, we provide a detailed description, supported by stepwise pictures and videos, of how to quickly and simply dissect the mouse ETA muscle for subsequent immunohistochemical analysis of muscle fibre types and NMJs. We also describe how ETA muscles can be imaged over short periods in live, anaesthetised mice to enable intravital NMJ assessment. Finally, by dissecting muscles from young adult males, we provide reference data on ETA fibre types in C57BL6/J mice.

## 2 MATERIALS AND METHODS

### 2.1 General

Mouse handling and experiments were performed under licence from the U.K. Home Office in accordance with the Animals (Scientific Procedures) Act (1986) and approved by the University College London—Queen Square Institute of Neurology Ethics Committee. Both male and female mice on the C57BL6/J background were used, ranging in age from postnatal Day 5 (P5) to P341 (detailed in figure legends). Hemizygous B6. Cg‐Tg(Thy1‐CFP/COX8A)S2Lich/J mice (Thy1‐mitoCFP, IMSR Cat# JAX:007967, RRID:IMSR_JAX:007967) were used for live imaging of neuronal mitochondria expressing cyan fluorescent protein (CFP) (Misgeld et al., 2007).

### 2.2 Reagents, equipment and set‐up

All of the equipment required for wholemount muscle dissection have been described previously (Sleigh et al., 2014a). These or similar tools can also be used to prepare the ETA for in vivo imaging, while a description of how to induce anaesthesia and perform intramuscular injections has been detailed elsewhere (Sleigh et al., 2020b; Turney et al., 2012). Information on the primary and secondary antibodies used for immunofluorescence are provided in Tables 1 and 2, respectively. AlexaFluor 488 and 555 α‐bungarotoxin (α‐BTX, Life Technologies, B13422 and B35451/RRID:AB_2617152, respectively, 1:1000) were used to identify postsynaptic acetylcholine receptors (AChRs). The binding fragment of tetanus neurotoxin (H_C_T) was produced and labelled with AlexaFluor 555 C_2_ maleimide (Life Technologies, A20346) as previously described (Gibbs et al., 2016). All dissection images and videos were taken using a DSK 500 dual head stereo microscope (Motic, Barcelona, Spain, PM5539B901) with attached Moticam 1080 HDMI digital camera (Motic, MC1080). Fixed and live immunofluorescent images were taken on an inverted LSM780 laser scanning microscope (Zeiss) using a 20×, 40× or 63× objective.

**TABLE 1:** Primary antibodies used in this study

**Target**	**Species**	**Isotype**	**Company**	**Catalogue #**	**RRID**	**Dilution**
Laminin	Rb	IgG	Sigma	L9393	AB_477163	1:250
Myosin heavy chain type I	Ms	IgG2b	DSHB	BA‐D5	AB_2235587	1:100
Myosin heavy chain type IIA	Ms	IgG1	DSHB	SC‐71	AB_2147165	1:100
Myosin heavy chain type IIB	Ms	IgM	DSHB	BF‐F3	AB_2266724	1:100
Neurofilament (2H3)	Ms	IgG1	DSHB	2H3	AB_531793	1:250
Pan‐synaptic vesicle 2 (SV2)	Ms	IgG1	DSHB	SV2	AB_2315387	1:50

Abbreviations: DSHB, Developmental Studies Hybridoma Bank; Ms, mouse; Rb, rabbit.

**TABLE 2:** Fluorescent secondary antibodies used in this study

Target	Fluorophore	Species	Company	Catalogue #	RRID	Dilution
Rabbit IgG	AlexaFluor 405	Gt	Life Technologies	A‐31556	AB_221605	1:500
Mouse IgG	AlexaFluor 488	Dk	Life Technologies	A‐21202	AB_141607	1:500
Mouse IgG2b	AlexaFluor 488	Gt	Life Technologies	A‐21141	AB_141626	1:500
Mouse IgM	AlexaFluor 568	Gt	Life Technologies	A‐21043	AB_2535712	1:500
Mouse IgG1	AlexaFluor 647	Gt	Life Technologies	A‐21240	AB_141658	1:500

Abbreviations: Dk, donkey; Gt, goat.

### 2.3 Muscle fibre analyses

Post‐extraction, nonfixed ETA muscles were embedded in 10 × 10 × 5 mm biopsy cryomolds (Sakura Finetek, 4565) containing optimal cutting temperature (OCT) compound (Sakura Finetek, 4583); care was taken to ensure that the ETAs were as straight and flat as possible before freezing on dry ice; 30‐μm sections, perpendicular to fibre length, were then cut throughout the muscle using an OTF Cryostat (Bright Instruments) and collected on polysine‐coated slides (VWR, 631‐0107). Slides were air dried for 30–60 min before staining or storage at −20℃. Muscle sections were stained as previously described (Rossor et al., 2020), except that sections were incubated with primary antibodies (Table 1) overnight at room temperature and secondary antibodies (Table 2) for 2 h at room temperature. Four sections per ETA were imaged at approximately equal positions throughout the muscle. Muscle fibre types and cross‐sectional areas were analysed using the MyoSight plugin for ImageJ (http://rsb.info.nih.gov/ij/) (Babcock et al., 2020). Alternative methods are also available (Mayeuf‐Louchart et al., 2018; Wen et al., 2018). Data were averaged across the four muscle sections to get values per animal.

### 2.4 NMJ staining

Following dissection and after cleaning away excess connective tissue, ETAs were stained in a 96‐well plate as previously detailed (Mech et al., 2020; Sleigh et al., 2014b).

## 3 ETA MUSCLE PROTOCOLS

### 3.1 Extraction

Animals should not be perfused with fixative as this prevents fibre‐type analysis and diminishes NMJ staining; however, perfusion with phosphate‐buffered saline (PBS) can aid dissection by preventing blood from pooling if the brachial artery is accidentally perforated. To preserve tissue health for downstream applications, all steps should be completed efficiently and tissue kept as cold as possible using ice‐cooled solutions.

**FIGURE 1:**
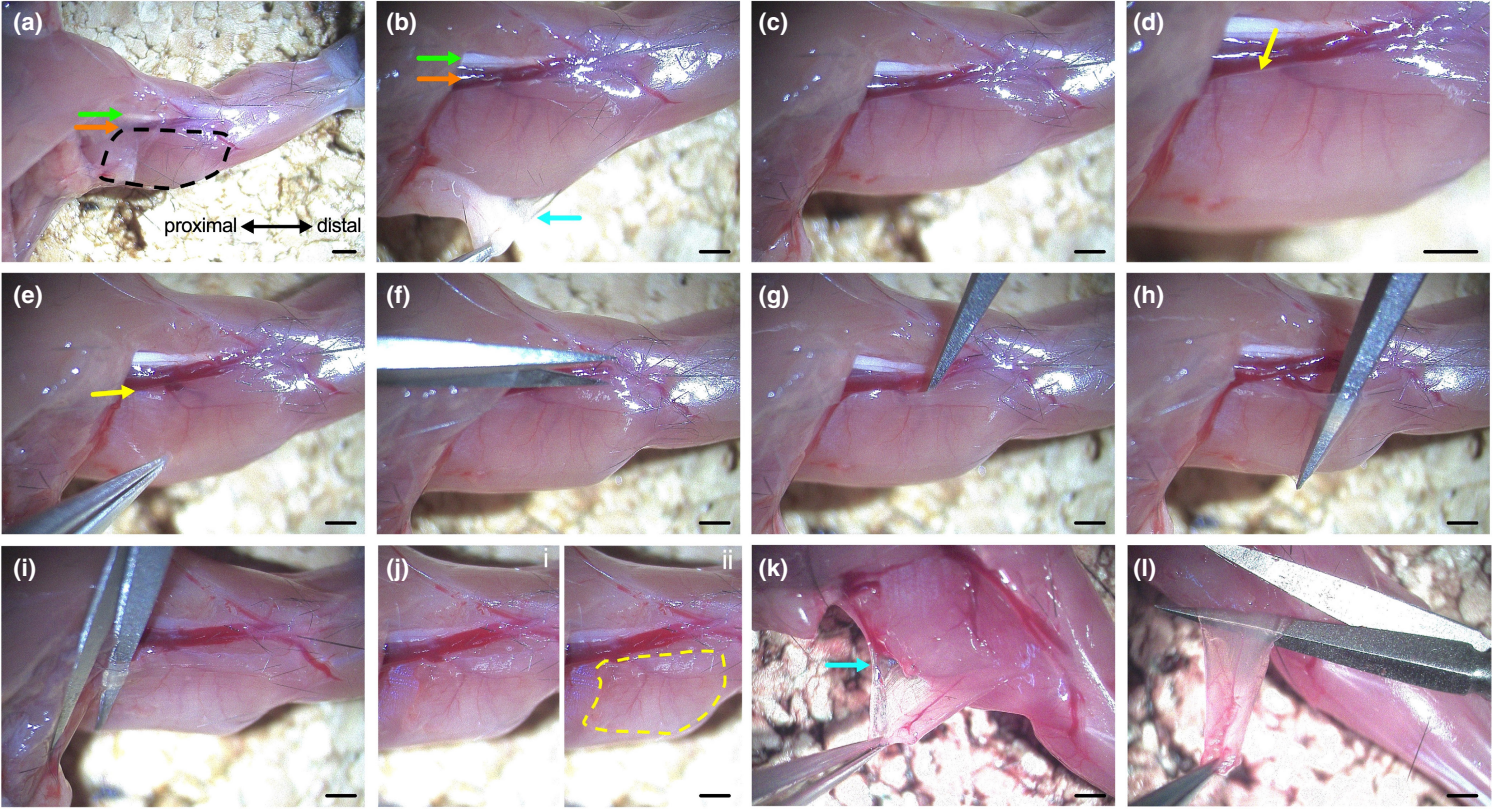
Dissection of the epitrochleoanconeus (ETA) muscle for neuromuscular junction (NMJ) and fibre‐type analyses. (a) The ETA (outlined by dashed black line) resides on the medial aspect of the upper forelimb, inferior to the median/ulnar nerves (green arrows) and brachial artery (orange arrows). The animal is ventral side upwards, and the wrist is to the right. (b,c) Grasp and blunt dissect away the connective tissue (cyan arrows) covering the muscle. (d) Locate the superior edge of the ETA (all yellow arrows), adjacent to the brachial artery. (e) Carefully grasping the inferior edge of the ETA can aid identification. (f) Cut through the connective tissue along the superior edge of the muscle, as well as the small blood vessel and nerve entering the ETA. Alternatively, to keep the nerve intact, see Figure S1. Do not cut the brachial artery at any point. (g) Place closed spring scissors beneath the ETA and gently push the blades through the connective tissue and out the other side of the muscle. (h) Move the blades from left to right to further tease the muscle from underlying tissues. (i) Cut through the proximal ETA, adjacent and parallel to the site of insertion. (j) After cutting, the ETA will remain in place (left panel *i*). Panel *ii* (right) is the same image as panel *i* but with the ETA outlined by a dashed yellow line. (k) Peel the ETA back distally, cutting and blunt dissecting the underlying connective tissue. (l) Cut through the distal edge of the muscle. Pictures were taken of a P37 male. Scale bars ≈ 1 mm. See also Figure S1 and Videos S1 and S2

Once the animal has been humanely euthanised, douse the upper body with ethanol to restrict fur dispersal and remove the pelt of the forelimb down towards the paw (Figure 1a). Place the animal ventral side up on a cork board and pin out the paw at a right angle to the body. Arising from the tendon of insertion of the latissimus dorsi (close to the pectoral muscles) and inserting into the medial epicondyle of the humerus (elbow area), the ETA is found inferior to the ulnar nerve and brachial artery (Figure 1a). Remove the connective tissue and fat overlying the ETA by gently grasping with forceps at the muscle surface (Figure 1b,c). Locate the superior edge of the ETA (inferior to the brachial artery) (Figure 1d) and cut along the adjacent connective tissue and through the small blood vessel and nerve supplying the ETA (Figure 1e,f; for magnified image, see Figure S1e). Take particular care to avoid the brachial artery (orange arrows in Figure 1a,b), as damaging it will flood the preparation with blood, impeding dissection. Carefully separate the thin, sheet‐like ETA from underlying connective tissue and muscle by threading closed spring scissors beneath the superior edge of the ETA and out through the other side (Figure 1g) and then slowly moving left and right towards the ends of the muscle (Figure 1h). If the ETA blood vessel and nerve were not already cut, this process will do so. Once the ETA has been separated, remove the closed spring scissors from beneath the ETA and then put a single blade behind the muscle and move to the proximal end (Figure 1i). Cut through the ETA as close to the site of insertion as possible and peel the muscle back towards the paw, cautiously pulling or cutting connective tissue to facilitate this (Figure 1j,k). Finally, cut through the distal end of the ETA, close to the elbow (Figure 1l). For videos of this procedure, which can be completed in 2–3 min, see Videos S1 and S2.

If a larger section of nerve is required, for example, for ex vivo nerve‐muscle electrophysiology, a slightly alternative approach to the dissection can be followed (Figure S1). Instead of cutting through the nerve and blood vessel at the edge of the ETA as before (Figure 1e,f), leave them intact and instead sever the distal muscle end (Figure S1a). Fold the muscle backwards in the direction of the body and relieve the underlying connective tissue using spring scissors (Figure S1b). Now cut through the proximal end of the ETA and peel the muscle up in the rostral direction, again cutting at the connective tissue holding the muscle in place (Figure S1c,d). Turn the muscle back down and over so that it sits in its natural position—the ETA nerve and blood vessels can be observed approximately mid‐way along the top edge of ETA (Figure S1e). Finally, detach the blood vessels entering the ETA and then separate the ETA nerve from connected tissues back towards the radial nerve before cutting. The blood vessel and nerve enter the muscle within the top quadrant of fibres (Figure S1f).

At this point, the dissected ETA can be used to analyse muscle fibre types as described in Section 2.3. Alternatively, for assessment of NMJ morphology, further dissection is required as outlined in the next section.

### 3.2 Cleaning

**FIGURE 2:**
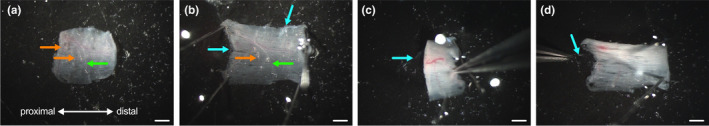
Cleaning of the epitrochleoanconeus (ETA) for immunofluorescent neuromuscular junction (NMJ) analyses. (a) Place the extracted muscle, either side up, into a Sylguard‐lined Petri dish filled with phosphate‐buffered saline (PBS). (b) Pin out the muscle using insect pins and fix for 10 min in 4% PFA in PBS. NMJs are generally found in the central third of the muscle (see Figure 5a), running from top to bottom in this image; thus, pinning the four corners of the muscle will not damage synapses. (c) Wash the muscle with PBS and then clean away as much connective tissue as possible without damaging the muscle fibres. Removing two pins and folding the ETA back on itself will aid identification of the connective tissue along the edge of the muscle. (d) Connective tissue and possibly some of the fascia can be observed and removed from the muscle edges. These latter steps will improve antibody penetration for successful NMJ staining. Pictures were taken of a P37 male. Nerves (green arrows), blood vessels (orange arrows) and connective tissue (cyan arrows) are highlighted. Scale bars ≈ 1 mm. See also Video S3

After extraction from the mouse, place the ETA in a Sylguard 184 silicone elastomer‐lined dish filled with PBS (Figure 2a). Carefully stretch and pin out the four corners of the muscle in any orientation using insect pins (Figure 2b). Fix the muscle using 4% paraformaldehyde (PFA, w/v, Thermo Scientific, 28908) in PBS for 10 min before washing with three dish‐volumes of PBS; this will increase muscle rigidity and make the connective tissue more visible. To improve antibody penetration for successful staining, remove as much superficial connective tissue as possible without causing damage to the fibres. This is done by cautiously grasping at the muscle surface to collect the transparent tissue. Two pins can then be removed and the muscle curled upwards to identify further connective tissue underneath the muscle (Figure 2c). Remove this and then repeat on the other side by turning the muscle over and repinning. Finally, remove any remaining connective tissue from the muscle borders (Figure 2d). For a recording of the post‐PFA cleaning procedure, which takes 3–5 min, see Video S3.

### 3.3 In vivo imaging

**FIGURE 3:**
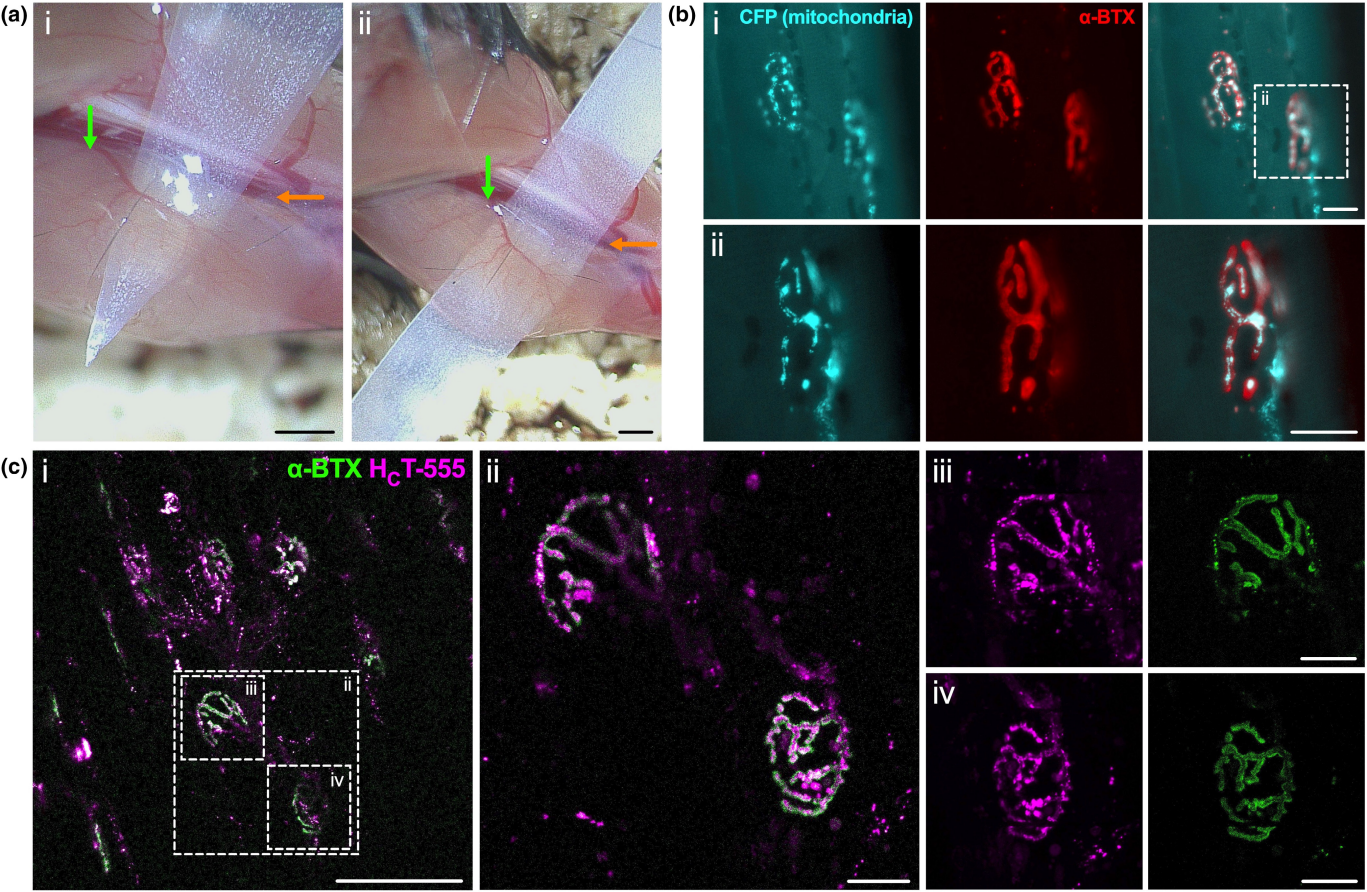
Live neuromuscular junctions (NMJs) from intact epitrochleoanconeus (ETA) muscles can be imaged in anaesthetised mice in vivo. (a) The ETA can be cautiously separated from underlying tissue in live, anaesthetised mice for intravital imaging. A long and thin piece of doubled‐up magic tape can then be placed under the distal portion of the muscle to aid imaging (panel *i*). When threading the magic tape underneath the muscle (panel *ii*), care must be taken to avoid cutting the blood vessel and nerve (green arrows) entering the ETA or damaging them with the edge of the magic tape. Similarly, perforation of the brachial artery (orange arrows) will be fatal. (b) Live NMJs can be imaged in real‐time in vivo. Fluorescent reporter strains, such as the Thy1‐mitoCFP ‘MitoMouse,’ can be used to identify NMJs and also increase the diversity of organelles that can be imaged at neuromuscular synapses. Mitochondria (cyan) are found at the axon terminal, co‐localising with α‐BTX (red). Prior to imaging, the ETA was bathed in α‐BTX for 1 min before washing thoroughly with phosphate‐buffered saline (PBS). The *ii* panels are magnified and focused images of the area within the dashed line box in panel *i*. (c) In addition to mitochondria, signalling endosomes that have taken up fragments of fluorescently labelled tetanus neurotoxin (H_C_T‐555) can be visualised at NMJs in vivo. H_C_T (magenta) was injected into the ETA 6 h prior to imaging, while α‐BTX (green) was topically applied for 1 min before washing and then imaging. Panels *iii* and *iv* are magnified images from panel *ii*, which is magnified from panel *i*. Pictures were taken of a P44 male (a), P170 female (b) and P341 female (c). N.b., the NMJs are all en face. Scale bars =1 mm (a), 20 µm (b,c *ii*–*iv*) and 100 µm (c *i*). All fluorescent images are single confocal planes. See also Figure S2

Induce anaesthesia using either isoflurane gas (Sleigh et al., 2020b) or ketamine/medetomidine injections paired with mechanical ventilation (Turney et al., 2012), as detailed elsewhere. Take particular care to ensure that the mouse lacks sensation before beginning the procedure and monitor the anaesthesia level throughout. Expose the ETA by removing the pelt of the upper arm, as performed when dissecting the muscle (Figure 1a). Continue by removing superficial connective tissue and fat covering the ETA (Figure 1b,c). On the side distal to the blood vessel and innervating nerve (i.e., towards the forepaw), place a pair of closed spring scissors or forceps underneath the ETA to separate it from the deeper tissues (Figure 1h). Carefully thread a thinly cut, doubled‐over piece of magic tape (Scotch, 3M) beneath the ETA (Figure 3ai) and pass it out through the other side (Figure 3aii). This separates the ETA from deeper tissues and ensures that it stays in contact with the coverslip when imaging. Cutting a point at the end of the tape can aid this process (Figure 3ai). Be wary not to damage the blood vessel or nerve entering the muscle (Figure S1e). At this point, the ETA can be bathed for 30–60 s in 5 µg/ml fluorescently labelled alpha‐bungarotoxin (α‐BTX) in PBS to identify postsynaptic AChRs close to the muscle surface (Figure 3b,c). If too concentrated, α‐BTX will block neuromuscular transmission and impact nerve function (Brown et al., 2014). After bathing, thoroughly wash the muscle with PBS to remove excess probe and then briefly dry the area with tissue. Move the mouse to the confocal microscope stage with prewarmed environmental chamber set to 37℃ to maintain body temperature. If using an inverted microscope (Sleigh et al., 2020b), then the arm of the mouse can be taped to the microscope stage insert with the ETA positioned on a glass coverslip above the objective. Prior to taping the arm, add a small amount of PBS to the coverslip to restrict desiccation and ensure that the magic tape is flat on the coverslip surface. Alternatively, an upright confocal equipped with suitable water immersion objectives can be used for live imaging of NMJs (Turney et al., 2012). Time lapse confocal imaging can then be performed on the ETA using fluorescent reporter strains such as the Thy1‐mitoCFP “MitoMouse” (Figure 3b). Organelle‐targeting fluorescent dyes and toxins can also be injected into the ETA a few hours prior to imaging (e.g., 2–6 h). For example, a fluorescently labelled, non‐toxic fragment of tetanus neurotoxin (H_C_T) can be injected; H_C_T binds to the NMJ and is internalised and sorted into signalling endosomes that are then retrogradely transported along axons to motor neuron cell bodies (Surana et al., 2018). Injections are performed on anaesthetised animals by making a small incision in the pelt above the ETA and administering substances with a pulled glass micropipette at a shallow angle to the muscle (~10–20°) (Figure S2) (Mohan et al., 2015). The wound is then closed by perpendicular stitching, using surgical suture, and the animal allowed to recover from anaesthesia. Given the superficial nature of the ETA, live imaging of NMJs in re‐anaesthetised animals can be achieved without two‐photon excitation microscopy.

## 4 RESULTS AND DISCUSSION

### 4.1 The ETA consists almost entirely of fast twitch fibres

**FIGURE 4:**
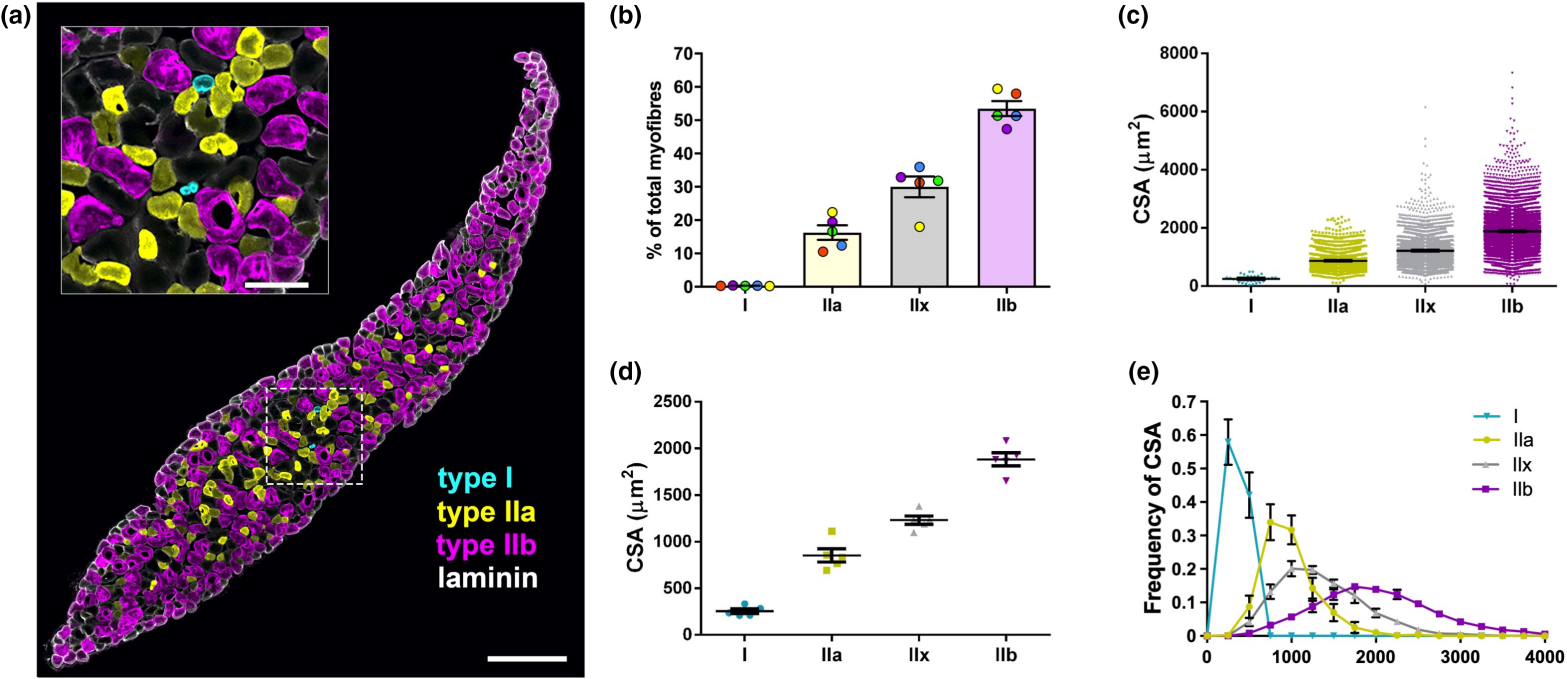
Epitrochleoanconeus (ETA) muscles are almost solely composed of fast‐twitch fibres. (a) Representative image of a 30‐µm transverse section cut through an ETA muscle. Type Ia fibres are stained cyan, type IIa in yellow and type IIb in magenta, while laminin (white) identifies the basement membrane surrounding individual fibres. Type IIx fibres are identified by a lack of myosin staining. The inset image (top left) is the magnified region within the dashed line box. Scale bars =300 µm (main) and 85 µm (inset). (b) Percentage of the total fibres corresponding to types I, IIa, IIx and IIb. The colours of individual data points identify the same animal. (c) Cross‐sectional area (CSA) of each fibre type. A total of 11,432 fibres were analysed. (d) Average CSA per animal for each fibre type. (e) Histogram of CSA frequencies by fibre type, binned in 250 µm^2^ intervals. For all panels, error bars represent standard error of the mean. P48 males were used. *n* = 5. All fluorescent images are single confocal planes

The mouse ETA has previously been described to possess 380 fibres with mean diameter of ~35 µm, ~90% of which are fast twitch fibres determined by ATPase histochemistry and myosin heavy chain analysis (Bradley et al., 1989). These data were reported in an abstract presented at the *Proceedings of the Physiological Society*, but the raw data and details about the mice (e.g., age and sex) are unavailable. We therefore dissected ETA muscles from five young adult males (P48) and subjected them to fibre‐type and cross‐sectional area analyses. We found that the mouse ETA muscle is composed of 571.6 ± 12.7 fibres, the vast majority of which correspond to type II fast twitch fibres (Figure 4a,b). At its widest point, the ETA is ~15 fibres thick and only ~2–4 at its thinnest. Intriguingly, every slice of muscle analysed exhibited only 1–3 type I fibres located near to the centre of the muscle (Figure 4a), equating to 0.28% ± 0.02% of the fibres (Figure 4b). Type IIa fibres tended to be located in the muscle interior and constituted 16.3% ± 2.2% of the fibres, whereas type IIx and type IIb were found throughout the muscle and made up 30.0% ± 3.1% and 53.5% ± 2.3% of the fibre population, respectively. Fibres at the muscle surface were almost exclusively type IIx and type IIb, indicating that the most superficial and therefore visually accessible NMJs are likely to preferentially innervate these fibre types.

We also analysed the cross‐sectional area of muscle fibres (Figure 4c) and the average cross‐sectional area per animal (Figure 4d), corroborating that type I fibres are smaller than type II fibres (Stifani, 2014). The distribution of fibre size is also presented as a histogram (Figure 4e); every type I fibre was smaller than 500 µm^2^, while type IIb fibres are larger than type IIa fibres, with type IIx showing an intermediate size distribution between types IIa and IIb.

It is unclear why our data on ETA muscle fibre numbers and types differ from those previously reported by Bradley et al., (1989). It may be due to differences in mouse age, sex or strain or perhaps the protocol used for fibre typing, although we cannot be sure, given the lack of data in the initial report. Nevertheless, we present reference data on ETA muscle fibres from young adult male C57BL6/J mice and show how the ETA could be used to analyse the impact of ageing or disease on fibre type proportions and cross‐sectional areas.

### 4.2 Degenerative and developmental NMJ phenotypes can be assessed in ETA muscle

**FIGURE 5:**
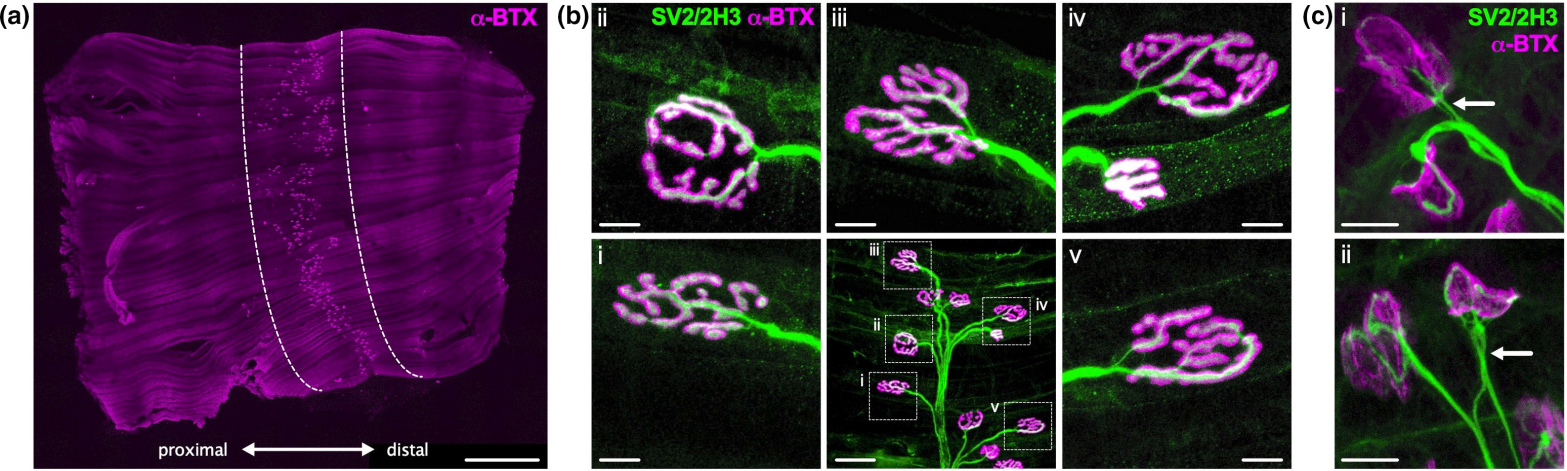
Neuromuscular junctions (NMJs) from dissected epitrochleoanconeus (ETA) muscle can be visualised to analyse nerve‐muscle connectivity and developmental phenotypes. (a) The central third of the ETA, perpendicular to muscle fibres, contains all NMJs. Alpha‐bungarotoxin (α‐BTX, magenta) was used to identify post‐synaptic AChRs. (b) Wholemount staining allows for easy imaging and assessment of many NMJs. Motor neurons are labelled with antibodies against synaptic vesicle 2 (SV2) and neurofilament (2H3). NMJs are identified by the overlapping staining between SV2/2H3 (green) and AChRs (magenta). Panels *i*–*v* are magnified images from the dashed line boxes in the bottom row, middle panel. (c) In addition to being able to assess denervation, NMJ labelling can be used to identify developmental processes such as synapse elimination and the plaque‐to‐pretzel transition of the postsynapse. Arrows highlight poly‐innervated NMJs that are yet to undergo synapse elimination to become innervated by a single motor neuron. Pictures were taken of P57 (a,b) and P5 (c) females. All images are maximum intensity z‐projections. Scale bars =1 mm (a), 50 µm (b bottom row, middle panel) and 10 µm (b *i*–*v*, c)

Once dissected and processed, motor neurons can be visualised using a combination of anti‐SV2 and anti‐2H3 antibodies to co‐stain the presynaptic motor terminal and axonal neurofilaments, respectively, while fluorescent α‐BTX will identify postsynaptic AChRs (Figure 5). Additional NMJ‐resident proteins can be imaged, including neurotrophic factors and their receptors (e.g., BDNF, TrkB and p75^NTR^), terminal Schwann cell proteins (e.g., S100B), presynaptic constituents required for neurotransmission (e.g., synaptophysin, synaptotagmin and bassoon), vascular proteins (e.g., Pecam1 and VEGFR2), postsynaptic elements (e.g., MuSK and rapsyn) and basement membrane proteins (e.g., nidogens) (Barik et al., 2014; Bercsenyi et al., 2014; Gonzalez et al., 1999; Liu et al., 2013; Pérez et al., 2019; Sleigh et al., 2017a; Spaulding et al., 2016; Tejero et al., 2016).

In healthy muscles, there is a one to one ratio between fibres and neuromuscular synapses; thus, there are ~570 NMJs that can be imaged per ETA (Figure 5a,b). In neuromuscular disease models, the contiguity between pre‐ and postsynaptic staining can be assessed as a measure of peripheral motor neuron degeneration (Ling et al., 2012; Murray et al., 2008; Williamson et al., 2019). Loss of neuromuscular connectivity, a process known as denervation, has occurred when the overlap between neuronal and AChR staining is reduced or completely absent. Accordingly, NMJs can be categorised as fully innervated, partially denervated or fully denervated/vacant (Sleigh et al., 2014a). In addition to studies of synaptic degeneration (Nguyen et al., 2012), the ETA has been used to monitor NMJ development, including the processes of synapse elimination and plaque‐to‐pretzel endplate transition (Figure 5c) (Rodríguez Cruz et al., 2020), as well as terminal sprouting in response to denervation (Nguyen et al., 2012; Rogozhin et al., 2008). While most of these processes can be scored by eye, ImageJ‐based tools, such as NMJ‐morph and NMJ‐Analyser (Jones et al., 2016; Mejia Maza et al., 2021; Minty et al., 2020), provide a more objective and systematic method for analysing NMJs in a quantitative manner, which can facilitate the identification of more subtle morphological phenotypes; for example, NMJ‐Analyser was used to identify structural defects in motor nerve terminals of two different ALS mouse models at a time when no clear denervation was detectable by eye (Mejia Maza et al., 2021).

### 4.3 ETA NMJs can be imaged in vivo

By cautiously separating the ETA from underlying tissues and placing magic tape beneath the muscle (Figure 3a), NMJs in live anaesthetised mice can be imaged in real time (Figure 3b,c). Briefly bathing the muscle in PBS containing fluorescently labelled α‐BTX allows visualisation of postsynaptic AChRs at the muscle surface. Given its orientation and flatness, the majority of ETA NMJs are en face, meaning that the largest aspect of the synapse and its postsynaptic perforations can be observed. This is particularly important for intravital imaging, as en face NMJs can be rapidly imaged using a single confocal plane, without needing to take time‐consuming z‐stacks. Here, we identified and time‐lapse imaged NMJs from Thy1‐mitoCFP mice, which express CFP in the mitochondria of neurons. Labelled mitochondria were found within α‐BTX‐stained areas, consistent with localisation at the motor nerve terminals (Figure 3b). To analyse signalling endosomes at the NMJ and within axons, we also trialled injection of a fluorescent retrograde probe (H_C_T) into the ETA prior to imaging (Figure S2). Like the CFP‐positive mitochondria, H_C_T‐positive puncta were found coincident with α‐BTX fluorescence, indicating that the probe had been successfully internalised into motor neurons (Figure 3c).

These experiments showcase the feasibility of using the ETA for intravital experiments that assess the real‐time kinetics of varied organelles at the NMJ and within intramuscular axons. While we have only attempted imaging of mitochondria and signalling endosomes, an array of alternative tools are available for live tracking of neuronal organelles and cargoes (Surana et al., 2020). Through ETA injection, the effect of drugs and treatments on cargo transport may be interrogated, as may the impact of nerve crush or transection; however, a high‐powered dissection scope and great care will be needed to avoid the ETA blood supply when attempting the latter (Figure S1e). We have only performed terminal imaging of the ETA (i.e., the mouse, although alive when being imaged, will be culled before the anaesthetic wears off); nevertheless, we believe that repeated imaging over time (i.e., with recovery between sessions), as has been performed on the sternomastoid and distal leg muscles, is a possibility. This would be significantly facilitated by using genetically encoded fluorescent strains, such as the Thy1‐YFP mouse that expresses yellow fluorescent protein (YFP) in motor and sensory neurons (Feng et al., 2000) or S100B‐YFP mice possessing fluorescent terminal Schwann cells (Zuo et al., 2004).

### 4.4 Troubleshooting

While dissecting the ETA, perforation of the brachial artery will result in blood flooding the dissection area. To avoid this, mice can be PBS‐ or saline‐perfused prior to dissection (Gage et al., 2012). Once the dissection technique is mastered, there will be less need for perfusion. Additionally, at first, it can be tricky to differentiate the thin ETA from deeper tissues. To aid this, softly grasp and pull the ETA or carefully drag forceps down the muscle (Figure 1e)—this will cause the ETA to move and thereby aid identification of the superior edge of the muscle.

Another issue encountered when processing the ETA for NMJ analyses is that the intensity of immunofluorescence may be low. Three simple steps to avoid this are (1) complete the dissection to fixation protocol as quickly as possible; (2) use freshly diluted, high quality PFA; and (3) fix the muscle for no longer than 10 min. If this does not improve staining, then there is likely an issue with clearing of the connective tissue and muscle fascia (Figure 2, Video S3). To improve antibody penetration, some of the fascia can be cautiously torn and removed from one side of the muscle. To identify the fascia, individual fibres can be teased using forceps at the distal and proximal ends of the muscle, away from the central band of NMJs (Figure 5a). This is more pertinent in older mice, where the fascia and connective tissues can thicken and becomes less elastic (Zullo et al., 2020). Take care during this procedure to limit damage to the underlying muscle fibres. Additionally, applying pressure to fixed wholemount diaphragm muscle has been shown to improve motor neuron staining, albeit with associated alterations in neuromuscular architecture (Tu et al., 2020). Alternatively, if issues with antibody penetration persist, transgenic mice with fluorescent motor neurons can be used (Lichtman & Sanes, 2003).

While preparing the ETA for intravital imaging, great care must be taken to avoid severing any blood vessels and damaging the innervating nerve. Before attempting the intravital procedure, we therefore suggest that dissection of the ETA is mastered using euthanised mice. Understanding the neuroanatomy and vasculature of the upper forelimb will also help with this (Greene, 1935).

Common issues with intravital imaging include image distortion through movement (e.g., breathing and muscle twitching), drop in body temperature, photobleaching/loss of fluorescence intensity and tissue phototoxicity (Turney & Lichtman, 2008). To reduce movement artefacts when using gaseous anaesthesia, the forearm is immobilised on the microscope stage using masking tape. It will take practice to find the optimal orientation with each individual microscope and anaesthetic machinery. Alternatively, ketamine/medetomidine can be used to anaesthetise the animal and depress respiration (Turney et al., 2012). The mechanical ventilation required to keep the animal alive can then be turned off for short periods to obtain distortion‐free images; however, this is not feasible for tracking organelle over periods longer than 30–60 s. Anaesthesia impairs normal thermoregulatory control, so ETA exposure for live imaging experiments must be performed on a prewarmed heating mat. Similarly, the confocal microscope requires an environmental chamber prewarmed to 37℃ to maintain body temperature. To limit photobleaching and possible phototoxicity, laser power should be kept to a minimum, the imaging period and frequency should be sensibly chosen and resulting videos monitored for signs of damage and instability. Finally, if using α‐BTX to visualise AChRs, avoid concentrations that can impair neurotransmission (Brown et al., 2014).

## 5 LIMITATIONS

The ETA can be dissected and processed for imaging from pups in the first week postbirth until late adulthood and old age. However, intravital imaging before P14 is difficult due to animal size and issues with anaesthesia. Furthermore, the in vivo NMJ assessment requires a high‐powered confocal microscope with temperature‐controlled environmental chamber and customised microscope stage.

## 6 CONCLUSION

Aided by images and videos (Figures 1 and 2, Videos [Link], [Link], [Link]), we provide a detailed protocol of how to rapidly dissect the mouse ETA muscle, which can then be used for a variety of neuromuscular analyses, including studies of muscle fibre type composition (Figure 4), as well as assessment of motor neuron development and degeneration/regeneration (Figure 5). These evaluations can be combined with RNA and protein analyses, electrophysiological recordings and possibly ex vivo synaptic degeneration assays (Dissanayake et al., 2020), to name but a few relevant applications, in order to interrogate rodent models of neuromuscular disorders. Furthermore, through comparison with other muscles, a detailed understanding of neuropathology and pertinent features of disease can emerge, especially when correlated with baseline properties of healthy muscles. We also present a description of how to image the ETA in live anaesthetised mice (Figure 3). This protocol and its adaptations have the potential to provide insights into temporal NMJ morphology, as well as organelle trafficking, and how these properties are impacted by disease. Moreover, the ETA is a good candidate for in vivo electroporation‐mediated gene transfer experiments to study NMJ protein function (Ojeda et al., 2020). Again, live comparison with other muscles, such as the sternomastoid, may highlight previously unappreciated features of dynamic processes occurring at the neuromuscular synapse. With the help of the techniques outlined here, we hope that the ETA will become a part of the experimental arsenal for assessment of neuromuscular pathology and live imaging of NMJ kinetics.

## CONFLICT OF INTEREST

The authors have no competing interest to declare.

## AUTHOR CONTRIBUTIONS

D.V.‐C., G.S. and J.N.S. performed the research; D.V.‐C. and J.N.S. wrote the manuscript; all authors approve submission of this work.

## Supporting information

Supplementary Material

Video S1

Video S2

Video S3

## Data Availability

The data that support the findings of this study are available from the corresponding author upon reasonable request.
