# Supplementary material for: Dissection, in vivo imaging and analysis of the mouse epitrochleoanconeus muscle
Source: J Anat. 2021 Jun 13;241(5):1108–19. doi: 10.1111/joa.13478 (PMC9558155; doi:10.1111/joa.13478)
Supplement: Supplementary file 1 — Supplementary Material [file JOA-241-1108-s004.pdf]

Dissection, *in vivo* imaging and analysis of the mouse epitrochleoanconeus muscle

Running Title: Mouse ETA Muscle Dissection

David Villarroel-Campos[1], Giampietro Schiavo[1,2] and James N. Sleigh[1,2,*]

[1] Department of Neuromuscular Diseases, UCL Queen Square Institute of Neurology, University College London, London WC1N 3BG, UK.

[2] UK Dementia Research Institute, University College London, London WC1E 6BT, UK.

ORCID:

Villarroel-Campos (0000-0003-4250-7216)

Schiavo (0000-0002-4319-8745)

Sleigh (0000-0002-3782-9045)

[*] Correspondence to James N. Sleigh: j.sleigh@ucl.ac.uk

Tel: +44(0)20 3448 4112

Fax: +44(0)20 7813 3107

Supplementary Information

Supplementary Figures and Legends

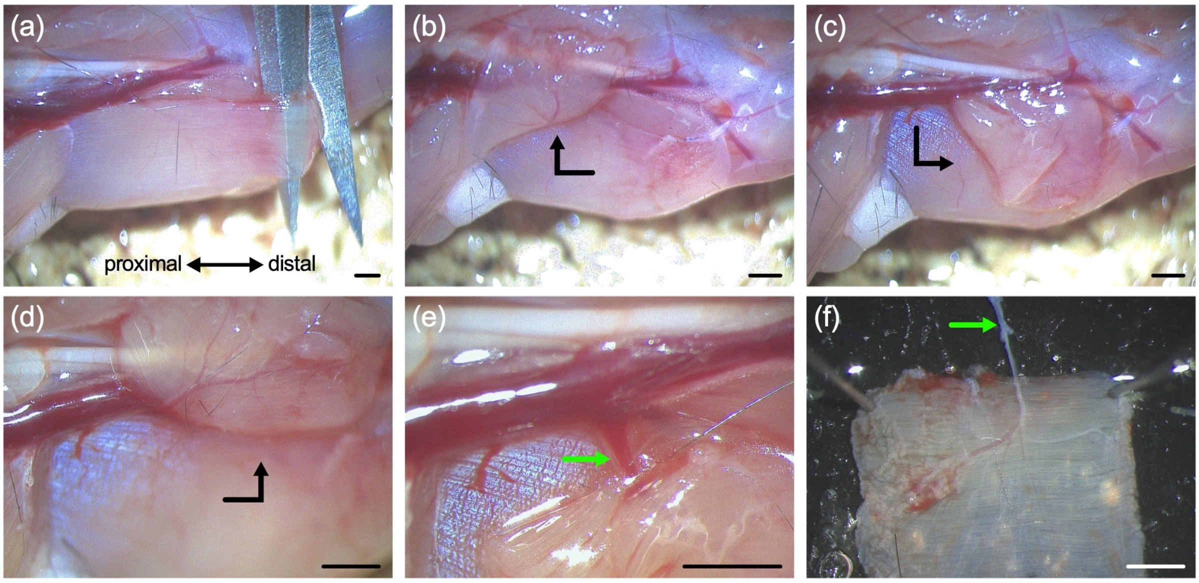

Supplementary Figure S1. An alternative ETA dissection protocol for nerve extraction. (a) When first separating the ETA, instead of cutting through the blood vessel and nerve (**Figure 1e-f**), place spring scissors beneath the muscle and cut through its distal end. **(b)** Fold the muscle back proximally (arrow) and cut through the connective tissue. **(c)** Cut through the proximal end of the muscle and fold back on itself distally (arrow), again pulling away connective tissue. **(d)** Fold the muscle upwards (arrow) and remove any remaining connective tissue. **(e)** Manoeuvre the muscle back into its natural orientation to observe the nerve innervating the ETA (green arrow) with adjacent blood supply. **(f)** Cut the blood vessel and then carefully trace the nerve back to the radial nerve by cutting away and blunt dissecting the attached connective tissue. A few millimetres of nerve can be readily obtained (green arrow). Pictures were taken of a P65 male. Scale bars ≈ 1 mm. See also **Figure 1**.

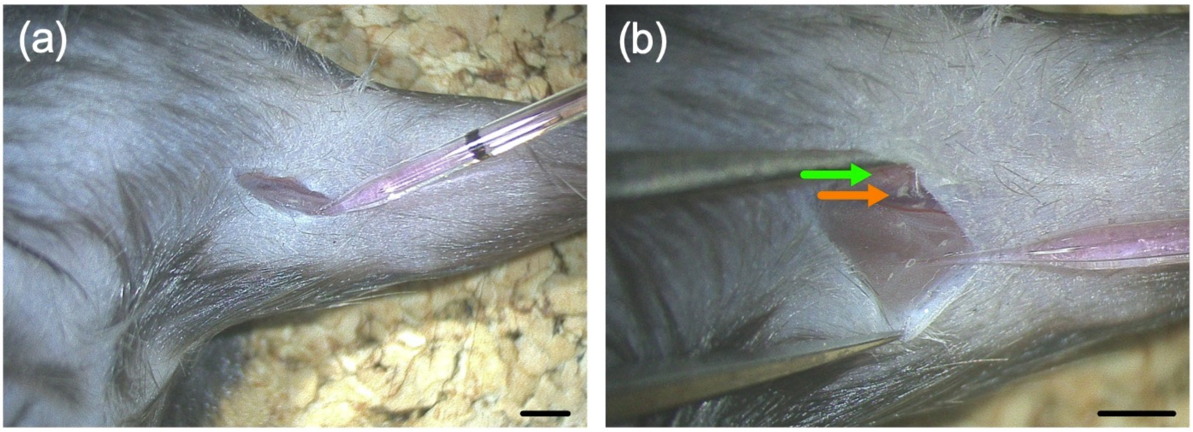

Supplementary Figure S2. Intravital injection of the ETA. (a) Under isoflurane anaesthesia, a small incision can be made in the pelt overlaying the ETA. Substances, such as drugs and fluorescent tracers (e.g., HcT), can then be injected into the muscle using a pulled glass micropipette. To reduce contamination with fur, the area can be shaved and dampened with ethanol prior to cutting. **(b)** The median/ulnar nerves (green arrow) and brachial artery (orange arrow) provide a useful point of reference for the ETA. After injecting the ETA, the skin incision can be sutured closed and the animal allowed to recover before subsequently re-anaesthetising for live imaging. Alternatively, if the desired effect of the injected substance occurs sufficiently quickly, the animal can remain anaesthetised from injection to imaging. Pictures were taken of a P65 male. Scale bars ≈ 1 mm. See also **Figure 3**.

Supplementary Video Legends

Supplementary Video S1. Left ETA dissection. This video shows extraction of the mouse ETA muscle from the left forelimb. First, some of the fat and connective tissue covering the ETA is removed by blunt dissection (starting at 00:01). The superior edge of the ETA is located (00:15) and some connective tissue along this edge is cut (00:19). Closed spring scissors are then placed beneath the ETA (00:21) before being gently pushed through the connective tissue and out the other side of the muscle (00:26). This latter motion detached the nerve and blood vessel supplying the ETA. More connective tissue on the superior edge of the muscle is pushed to the side (00:29) before the scissors are placed back beneath the ETA and pushed through to the other side two more times (00:32 and 00:42). The proximal edge of the ETA, adjacent and parallel to the site of insertion, is then cut (00:58). The ETA is peeled back distally, cutting and blunt dissecting the underlying connective tissue (01:05). Finally, the distal edge of the ETA is cut (01:46). A P37 male is presented in this video. See also **Figure 1**, which can be used to discern scale. Video run time is 01:53 and there is no audio.

Supplementary Video S2. Right ETA dissection. This video shows extraction of the mouse ETA muscle from the right forelimb. Fat and connective tissue covering the ETA are first removed (starting at 00:04). Extraneous muscle obscuring the ETA can be detached if necessary (00:25). The superior edge of the ETA is identified and the connective tissue along this edge is cut (00:40). Closed spring scissors are gently pushed through the connective tissue underlying the ETA and out the other side of the muscle (00:50). The proximal edge of the ETA, adjacent and parallel to the site of insertion, is then cut (01:22). The ETA is peeled back distally, cutting and blunt dissecting the underlying connective tissue (01:32). The nerve and blood vessel supplying the ETA are cut during this process (01:43). Finally, the distal edge of the ETA is cut (02:10). A P37 male is presented in this video. See also **Figure 1**, which can be used to discern scale. Video run time is 02:16 and there is no audio.

Supplementary Video S3. ETA cleaning for immunofluorescent analysis of NMJs. This video shows the process of removing superficial connective tissue from the ETA. After the short fixation step, two pins are removed from one edge of the ETA (starting at 00:01). Superficial connective tissue is then removed from the muscle edges (00:09) and on the flat side facing the Sylguard (00:21). The remaining pins are then removed (01:14) so that connective tissue can also be removed from the other side of the muscle. A P37 male is

presented in this video. See also **Figure 2**, which can be used to discern scale. Video run time is 02:15 and there is no audio.
